# Modification of Seed Oil Composition in *Arabidopsis* by Artificial microRNA-Mediated Gene Silencing

**DOI:** 10.3389/fpls.2012.00168

**Published:** 2012-07-31

**Authors:** Srinivas Belide, James Robertson Petrie, Pushkar Shrestha, Surinder Pal Singh

**Affiliations:** ^1^Food Futures National Research Flagship, CSIRO Plant IndustryCanberra, ACT, Australia; ^2^Department of Biotechnology, Sreenidhi Institute of Science and TechnologyHyderabad, Andhra Pradesh, India

**Keywords:** miR159b, fatty acid desaturase 2, fatty acid elongase, fatty acyl-ACP thioesterase B, fatty acid profiles

## Abstract

Various post transcriptional gene silencing strategies have been developed and exploited to study gene function or engineer disease resistance. The recently developed artificial microRNA strategy is an alternative method of effectively silencing target genes. The Δ12-desaturase (FAD2), Fatty acid elongase (FAE1), and Fatty acyl-ACP thioesterase B (FATB) were targeted with amiR159b-based constructs in *Arabidopsis* *thaliana* to evaluate changes in oil composition when expressed with the seed-specific *Brassica napus* truncated napin (FP1) promoter. Fatty acid profiles from transgenic homozygous seeds reveal that the targeted genes were silenced. The down-regulation of the *AtFAD-2* gene substantially increased oleic acid from the normal levels of ∼15% to as high as 63.3 and reduced total PUFA content (18:2^Δ9,12^ + 18:3^Δ9,12,15^ + 20:2^Δ11,14^ + 20:3^Δ11,14,17^) from 46.8 to 4.8%. Δ12-desaturase activity was reduced to levels as low as those in the null fad-2-1 and fad-2-2 mutants. Silencing of the FAE1 gene resulted in the reduction of eicosenoic acid (20:1^Δ11^) to 1.9 from 15.4% and silencing of FATB resulted in the reduction of palmitic acid (16:0) to 4.4% from 8.0%. Reduction in FATB activity is comparable with a FATB knock-out mutant. These results demonstrate for the first time amiR159b constructs targeted against three endogenous seed-expressed genes are clearly able to down-regulate and generate genotypic changes that are inherited stably over three generations.

## Introduction

MicroRNAs (miRNAs) are a class of 20–24-nucleotide (nt) regulatory small RNAs (sRNA) endogenous to both plants and animals which represent a recently developed miRNA-based strategy to silence endogenous genes. It has been demonstrated that the alteration of several nucleotides within the miRNA sequence does not affect its biogenesis as long as the positions of matches and mismatches within the precursor stem loop remain unaffected (Herve et al., 2004). This raises the possibility of modifying miRNA sequences and creating artificial miRNAs (amiRNA) directed against any gene of interest resulting in posttranscriptional silencing of the corresponding transcript. The amiRNA technology was first used for gene knock down in human cell lines, and it was successfully employed to down-regulate gene expression without affecting the expression of other unrelated genes in transgenic plants (Schwab et al., 2006; Warthmann et al., 2008). Since its successful application in silencing endogenous genes in plants several amiRNA vectors viz. ath-miR159a, ath-miR164b, ath-miR172a, ath-miR319a, and osa-miR528 recently been developed and tested.

Artificial microRNA based gene silencing is becoming a powerful tool and now commonly used in plant genetic engineering and under certain circumstances, can offer advantages over traditional hairpin-based RNA interference (RNAi). Foremost among these is the ability to silence specific transcripts amongst gene families with a reduced risk of cross-silencing and, because of this, amiRNA has been described as a second-generation RNAi technology (Tang et al., 2007). There are numerous examples of the use of amiRNA to down-regulate endogenous plant genes (Alvarez et al., 2006; Khraiwesh et al., 2008; Molnar et al., 2009) and also for developing transgenic virus resistance (Niu et al., 2006; Qu et al., 2007; Duan et al., 2008; Zhang et al., 2010; Ai et al., 2011). amiRNA has also been widely used for gene function studies and has moved rapidly from model plants to crop species (Gaurav et al., 2011), for instance in elucidating the function of FLOT4 and FLOT2 in nitrogen fixation in *Medicago truncatula* (Haney and Long, 2010). Male sterility has been achieved by targeting TBP-associated factors which plays crucial role in many developmental aspects including pollen development in the egg plant (Toppino et al., 2011).

Naturally occurring mutations in genes encoding fatty acid metabolizing enzymes provide a resource for novel oilseed phenotypes, but the effects of such changes are evident in all tissues and organs of the plant (Somerville and Browse, 1991). In contrast, transgenic gene suppression techniques in conjunction with seed-specific promoters has been used as a way to restrict the effects of targeted gene suppression to oil accumulating tissues within seeds (Pidkowich et al., 2007). Many attempts have been made to improve the fatty acid composition of plant oils through the seed-specific expression of various heterologous biosynthetic activities, with varying degrees of success (Napier, 2007) and tissue specific gene silencing is going play a major role in tailoring plant lipid composition. High oleic oils have real opportunity to substitute existing products used in high stability applications due to functional, health, and sensory benefits. Monounsaturates have higher rates of oxidative stability and consequently, have lower formation rates of oxidation products. In addition to food uses, high oleic oils have industrial applications (cosmetic, lubricant, transmission, and hydraulic) with possible ecological benefits through replacement of mineral oil. Also, saturated fatty acids such as palmitic acid (16:0) have been suggested to play a role in raising LDL cholesterol so oils that are engineered to be low in palmitic acid and rich in oleic acid could offer health benefits.

Several approaches, including antisense (Sellwood et al., 2000), co-suppression (Jadhav et al., 2005) and RNAi (Surinder et al., 2000), combined antisense-hairpin RNA (Tam and John, 2009) have been used to silence the gene expression related to oil synthesis in seed tissues. Importantly, there are examples of genetically modified soybean events with RNAi constructs that target FAD2 and/or Fatty acyl-ACP thioesterase B (FATB) genes for increased oleic acid accumulation that are being deregulated in several countries (e.g., 305423 and MON87705 high oleic soybeans). However microRNA based gene silencing has been not reported in model plants or other economically important oil seed crops for modulating the gene expression related to oil synthesis. In this paper we report for the first time the use of amiRNA based gene silencing of three important genes, i.e., Fatty acid desaturase 2 (FAD2), Fatty acid elongase (FAE1), and FATB involved in seed oil metabolism.

## Materials and Methods

### Construction of three artificial *A. thaliana* MIR159B genes for seed-specific plant expression

**Figure 1:**
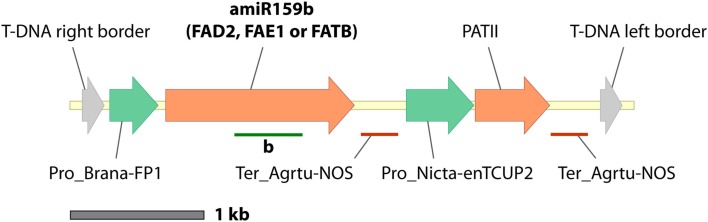
**Construct maps (T-DNA border-border regions only) the single-gene miR159b-based pJP1106, pJP1109, and pJP1110 which differ only in the 21mer sequences in the stem loops**. Stem loops are shown as green bars under the amiRNA genes.

**Figure 2:**
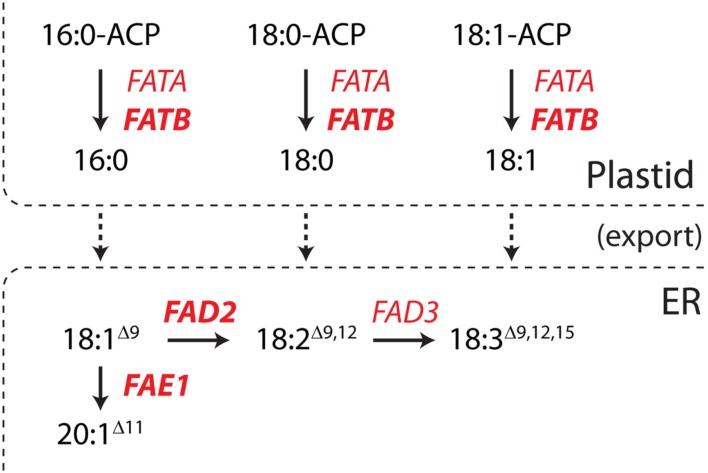
**A truncated fatty acid synthesis pathway depicting the roles of the enzymes described in this study**. The fatty acid desaturase 2 (FAD2), fatty acid elongase (FAE1), and fatty acyl-ACP thioesterase B (FATB) enzymes are in bold. In short, FATB is an acyl–acyl carrier protein thioesterase which primarily hydrolyzes saturated acyl-ACPs before the export of the fatty acid. 18:1^Δ9^, which is synthesized by other enzymes, can be either elongated by FAE1 or desaturated by FAD2.

A seed-specific expression binary vector, pJP1105 (GenBank accession JX155386), was constructed by cloning a modified version of the *Arabidopsis thaliana* miR159b gene from pBlueGreen (kindly supplied by Dr. Peter Waterhouse; Eamens et al., 2009) into the *Spe*I-*Xho*I sites of a vector, FP1-pORE03. This vector was constructed by cloning a truncated napin promoter (FP1) into binary vector pORE03 (Coutu et al., 2007). The CSIRO software package MatchPoint (http://www.pi.csiro.au/RNAi; Horn and Waterhouse, 2010) was used to identify three 21mer sequences which would target *A. thaliana* FAD2, FAE1, and FATB, respectively, without affecting off-target genes (Table 1). Three seed-specific expression versions of artificial *A. thaliana* ara-miR159b genes were generated by cloning modified stem loop regions into pJP1105 at the *Lgu*I sites to generate pJP1106, pJP1109, and pJP1110 (Figure 1). The genes targeted in these experiments were all fatty acid synthesis genes and were chosen since the phenotypes of lines in which they were silenced were quantifiable. FAD2 silencing results in increased 18:1^Δ9^, the substrate of the FAD2 enzyme. FAE1 silencing is most easily discerned by a decrease in the level of the product fatty acid, 20:1^Δ11^ whilst FATB silencing is most noticeable as a decrease in 16:0 which is exported to a lesser degree from the plastid to the cytosol (Figure 2).

**Table 1:** **Oligonucleotides used to modify miR159b stem loops in this study**.

Name	Oligonucleotide sequence
FAD2-F	TATATGCTCTTCGAGAGGGGCCTCGATGAGATGCCTCTTGGA
	GGGTTTAGCAGGGTGAAGTAAAG
FAD2-R	TATATGCTCTTCGAGATGGGCCTCGATGATCTGCCTCTAGAAG
	AGTGAAGCCATTAAAGGG
FAE1-F	TATATGCTCTTCGAGAGGACCGGAGACGGGACAAGTACTGGA
	GGGTTTAGCAGGGTGAAGTAAAG
FAE1-R	TATATGCTCTTCGAGATGACCGGAGACGGTCCAAGTACAGAAG
	AGTGAAGCCATTAAAGGG
FATB-F	TATATGCTCTTCGAGAGGACCTGGGTCAGGAAGTCTGGTGGA
	GGGTTTAGCAGGGTGAAGTAAAG
FATB-R	TATATGCTCTTCGAGATGACCTGGGTCAGTCAGTCTGGAGAAG
	AGTGAAGCCATTAAAGGG

*The 21mer regions targeting the *Arabidopsis thaliana* genes are underlined*.

### *Arabidopsis* plant transformation

pJP1106, pJP1109, and pJP1110 was transformed into separate *Agrobacterium tumefaciens* strain AGL1 by electroporation and the transformed strains used to introduce the genetic construct into *A. thaliana* (ecotype Columbia). Plant transformation was carried out using the floral dipping method (Clough and Bent, 1998). All transgenic plants were grown in a greenhouse under natural day length with a controlled temperature of 24°C in the daylight hours and 18°C in the evening, while Columbia wild type and hpRNA FAD2 silenced line (3-1-1; Stoutjesdijk et al., 2002) were grown alongside to compare the efficiency of silencing. Sterilized seeds (T_1_ seeds) from the transformed plants (T_0_ plants) were plated out on MS media (Murashige and Skoog, 1962) supplemented with 3.5 mg/L PPT and incubated at 24 ± 1°C under 16 photoperiod with the light intensity of 200 μmol/m^2^/s for 3 weeks. Surviving seedlings were subjected to another round of PPT selection with similar conditions for another 2 weeks and ∼30 PPT-resistant plants for each construct were isolated and transferred to soil to establish T_1_ transgenic plants. Most of these T_1_ plants were expected to be heterozygous for the introduced genetic construct. T_2_ seed from the transgenic T_1_ plants (selfed) were collected at maturity and analyzed for fatty acid composition. Lines displaying an apparent high level of FAD2 silencing were again germinated on PPT media and seedlings from lines with a 3:1 surviving:susceptible seedling ratio were transferred to soil. These lines were likely to contain single locus insertions and be either heterozygous or homozygous for the transgenic insert as null lines were selected against.

### Fatty acid analysis

Fatty acid methyl esters (FAME) were formed by transesterification of triplicate samples of ∼200 seeds each by heating with MeOH-CHCl_3_-HCl (10:1:1, v/v/v) at 90–100°C for 2 h in a glass test tube fitted with a Teflon-lined screw-cap. FAME were extracted into hexane:dichloromethane (4:1, v/v) and analyzed by GC. GC was performed as described by Zhou et al. (2006) but using a BPX 70 polar column (SGE, Ringwood, VIC, Australia). Relative fatty acid compositions were calculated as the percentage that each fatty acid represented of the total fatty acid profile. Alterations to the activity of the Δ12-desaturase caused by the action of introduced transgenes could be seen as changes in the amounts of oleic acid and in the seed oil profiles. An additional indirect method of assessing the cumulative effects of Δ12-desaturase activity during seed fatty acid synthesis is through the oleic desaturation proportion (ODP) parameter, derived by the following formula:

$$\text{ODP}=\frac{\text{\%18}:\text{2}+\text{\%18}:\text{3}}{\text{\%18}:1+\%18:2+\%18:3}$$

Oleic desaturation proportion represents the ratio of the total fatty acids accounting for the products of 18:1^Δ9^ desaturation (i.e., 18:2^Δ9,12^ and 18:3^Δ9,12,15)^ to the total amount of 18:1^Δ9^ substrate that was available, i.e., these products of 18:1^Δ9^ modification plus the remaining 18:1^Δ9^. *Arabidopsis* (ecotype Columbia) typically has an ODP value of around 0.70–0.79, indicating that around 70–79% of 18:1^Δ9^ formed during fatty acid synthesis is subsequently converted to the polyunsaturated C18 fatty acids initially via the action of Δ12-desaturase. This parameter is useful in illustrating the effects of *FAD2* gene silencing on the level of endogenous Δ12-desaturase activity.

Similarly, oleic elongation proportion (OEP) represents the ratio of the total fatty acids accounting for the products of 18:1^Δ9^ elongation (i.e., 20:1^Δ11^ and 22:1^Δ13^) to the total amount of 18:1^Δ9^ substrate that was available, i.e., the products of 18:1^Δ9^ modification (18:2^Δ9,12^ and 18:3^Δ9,12,15^) plus the remaining 18:1^Δ9^. *Arabidopsis* typically has an OEP value of around 0.20–0.22, indicating that around 20–22% of 18:1^Δ9^ formed during fatty acid synthesis is subsequently elongated via the action of FAE1. This parameter is useful in illustrating the effects of FAE1 gene silencing on the level of endogenous FAE1 activity.

$$\text{OEP}=\frac{\%20:1+\%22:1}{\%18:1+\%18:2+\%18:3+\%20:1+\%22:1}$$

### Statistical analysis

Quantitative data, fatty acid profiles from homozygous seeds (T_3_ and T_4_) were analyzed by One Way Analysis of Variance (ANOVA) using Sigmaplot (v12) by the Holm–Sidak method (Zar, 2010). Differences were considered significant at the 5% level.

## Results and Discussion

### Effect of gene silencing on Δ12 desaturation levels in T_2_ and T_3_ seed

**Figure 3:**
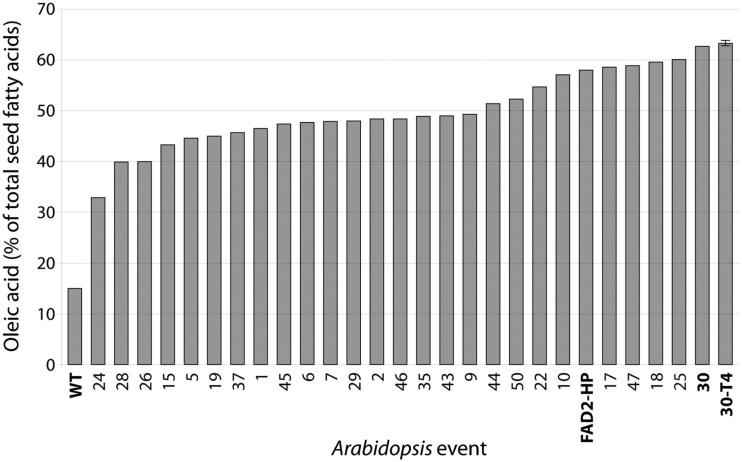
**Oleic acid content (%) of selfed seed of *Arabidopsis* FAD2 silenced line (T_2_, *n* = 30), line 30 forwarded to T_3_ and T_4_ generations**. The average T_4_ (*n* = 23) oleic acid content was also shown. Columbia (WT), FAD2 silenced line with RNAi approach (3-1-1) was also shown.

**Figure 4:**
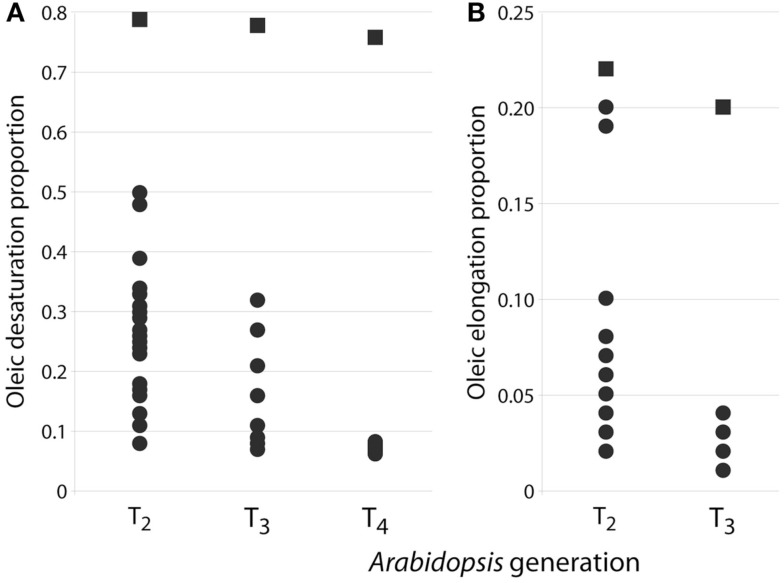
**(A)** Distribution of oleic desaturation proportion (ODP) values for FAD2 silenced T_2_ (*n* = 30), T_3_ (*n* = 21), and T_4_ (*n* = 23) seed (circles) in comparison with Columbia wildtype seeds (squares) which were grown alongside each generation. **(B)** Distribution of oleic elongation proportion (OEP) values for FAE1 silenced T_2_ (*n* = 11), T_3_ (*n* = 12), and Columbia control seeds.

FAD2 encodes an endoplasmic reticulum-localized Δ12-desaturase required for converting the monounsaturated oleic acid (18:1^Δ9^) to the polyunsaturated linoleic acid (18:2^Δ9,12^; Okuley et al., 1994). FAD2 is a classic reporter for silencing assays because it is a single-copy, non-essential gene in *Arabidopsis* and easily quantifiable (Miquel and Browse, 1992). Δ12-Desaturase is highly active in developing seeds of non-transgenic *Arabidopsis* (Columbia) and 18:1^Δ9^ is 12.9 ± 0.1 (Table 2). Levels of 18:1^Δ9^ in T_2_ seed transformed with the pJP1106 construct (FAD2 target) ranged from 32.9 to 62.7% in 30 independent transgenic events compared to an average non-transgenic parental level of 12.9 ± 0.1. A highly silenced line (plant ID-30, Figure 3) which has a single transgene insertion, determined by segregation ratios (3:1) of plant selectable marker (PPT) was forwarded to next generation (T_3_). Similarly high levels of the 18:1^Δ9^ were observed in T_3_ seed ranging from 46.0 to 63.8% with an average of 57.3 ± 5.0% in 21 independent transgenic events. In T_4_ seed also similar high level of 18:1^Δ9^ observed from 61 to 65.8% with an average of 63.3 ± 1.0% in 23 independent transgenic events. All the means were significantly different from all others (*P *< 0.01). The total PUFA content (18:2^Δ9,12^ + 18:3^Δ9,12,15^ 20:2^Δ11,14^ + 20:3^Δ11,14,17^) in T_2_ seed ranged from 6.1 to 38% but in homozygous lines the total PUFA content was further reduced and ranged from 4.3 to 5.7%. The control *Arabidopsis* ecotype Columbia has ODP value ranging 0.75–0.79, meaning that over 75% of oleic acid produced in the developing seed is subsequently converted to 18:2^Δ9,12^ and 18:3^Δ9,12,15^. The *fad-2-1* mutant has an ODP value of 0.17, indicating about a 75% reduction in Δ12 desaturation. The ODP values ranged from 0.08 to 0.48 in T_2_ seed, 0.07–0.32 in T_3_ seed, and 0.06–0.08 in T_4_ seed in contrast to 0.75 in control *Arabidopsis* ecotype Columbia (Figure 4A). The drastic reduction in ODP values in transgenic lines clearly indicates the efficient silencing of FAD2 gene using microRNA approach in the present study. Liu et al. (2002) and Maria et al. (2008) demonstrated the reduced levels of ODP are always associated with reduced desaturase mRNA levels in cotton and *Arabidopsis* using hpRNA and artificial trans-acting siRNA when compared with the control. The degree of FAD2 silencing and the amount of 18:1^Δ9^ (63.3 ± 1.1%) observed in this study using amiRNA is higher than the well characterized FAD-2-2 mutant (59.4 ± 2.0%), hairpin (56.9 ± 3.6%), and hairpin-antisense approach (61.7 ± 2.0%, Tam and John, 2009). The mean 18:2^Δ9,12^ + 18:3^Δ9,12,15^ in FAD2 silenced line using amiRNA is 4.7 ± 0.4% which is lower than the previously reported FAD-2-2 mutant (7.5 ± 1.1%) and FAD2 silenced line using hairpin-antisense approach (7.2 ± 1.4%; Tam and John, 2009) and also lower than the hpFAD-2 silenced line (8.3 ± 0.2%; Stoutjesdijk et al., 2002) compared in this study (Table 3).

**Table 2:** **Fatty acid profiles of *Arabidopsis thaliana* T_3_ homozygous seeds transformed with amiRNAs targeting FAE1 (pJ1109) and FATB (pJ1110), respectively**.

Fatty acids	Control	Silenced gene	
	Columbia	FAE1	FAT-B
**16:0**	**8.0 ± 0.3**	8.0 ± 0.2	**4.4 ± 0.5**
16:1^Δ9^	0.3 ± 0.1	0.1 ± 0.0	0.1 ± 0.1
18:0	3.1 ± 0.2	2.9 ± 0.3	2.0 ± 0.1
18:1^Δ9^	12.9 ± 0.1	24.0 ± 0.8	16.0 ± 0.8
18:1^Δ11^	1.9 ± 0.1	2.2 ± 0.1	1.6 ± 0.1
18:2^Δ9,12^	31.4 ± 0.6	34.9 ± 1.1	32.5 ± 0.5
18:3^Δ9,12,15^	19.6 ± 0.4	25.1 ± 0.9	23 ± 0.7
20:0	2.0 ± 0.0	0.4 ± 0.1	1.0 ± 0.1
**20:1^Δ11^**	**15.4 ± 0.3**	**1.9 ± 1.0**	15.2 ± 0.6
20:1^Δ13^	1.7 ± 0.1	0.4 ± 0.1	1.5 ± 0.2
20:2^Δ9,12^	1.8 ± 0.1	0.1 ± 0.0	1.4 ± 0.1
22:0	0.5 ± 0.1	0.1 ± 0.1	0.1 ± 0.0
22:1^Δ13^	1.3 ± 0.1	–	1.2 ± 1.2
24:0	0.1 ± 0.0	–	–

*Values are the means of the entire T_3_ seed of 21 plants (% of total fatty acids) with the errors reported as SD. Fatty acids directly affected by the gene silencing are shown in bold*.

**Table 3:** **Fatty acid profiles of *Arabidopsis thaliana* T_4_ homozygous seeds transformed with single-gene amiRNAs (pJP1106) targeting FAD2**.

Fatty acids	Control Columbia	FAD2 silenced line	hpRNA silenced line (3-1-1)
14:0	0.1 ± 0.0	0.1 ± 0.0	0.1 ± 0.0
16:0	6.8 ± 0.1	4.7 ± 0.1	4.9 ± 0.2
16:1^Δ9^	0.3 ± 0.0	0.3 ± 0.0	0.40 ± 0.0
16:3^Δ9,12,15^	0.1 ± 0.0	0.1 ± 0.0	0.1 ± 0.0
18:0	2.9 ± 0.0	2.8 ± 0.1	2.8 ± 0.1
**18:1^Δ9^**	**18.3 ± 1.0**	**63.3 ± 1.1**	**58.1 ± 1.1**
18:1^Δ11^	1.7 ± 0.1	2.1 ± 0.0	2.1 ± 0.2
18:2^Δ9,12^	27.5 ± 0.2	1.7 ± 0.2	3.3 ± 0.1
18:3^Δ9,12,15^	17.3 ± 0.9	3.0 ± 0.2	5.0 ± 0.1
20:1^Δ11^	17.6 ± 0.3	17.5 ± 0.7	18.5 ± 1.1
20:1^Δ13^	1.8 ± 0.1	1.7 ± 0.1	1.8 ± 0.1
20:2^Δ11,14^	1.6 ± 0.0	0.1 ± 0.0	0.1 ± 0.0
20:3^Δ11,14,17^	0.4 ± 0.0	0.0 ± 0.0	0.1 ± 0.0
20:0	1.7 ± 0.1	1.1 ± 0.2	1.1 ± 0.7
22:0	0.3 ± 0.0	0.3 ± 0.0	0.3 ± 0.0
22:1^Δ13^	1.5 ± 0.1	0.8 ± 0.1	0.9 ± 0.1
24:0	0.2 ± 0.0	0.2 ± 0.0	0.2 ± 0.0
24:1^Δ15^	0.2 ± 0.0	0.2 ± 0.0	0.2 ± 0.0

*Control Columbia and FAD-2-HP silenced line (Stoutjesdijk et al., 2002) profile were also shown. Values are the means of the entire T_4_ seeds of 23 independent plants (% of total fatty acids) with the errors reported as SD. Fatty acids directly affected by the gene silencing are shown in bold*.

Stability of inheritance was studied in single highly silenced FAD2 line up to three generations and ODP levels analyzed at each generation (Figure 4A). The wide variation of ODP levels in T_2_ seeds reflect different silencing efficacy of various transgene insertion events. The ODP of T_3_ and T_4_ seeds were significantly different from control Columbia and reflected the stability of the silencing over multiple generations.

### FAE1 gene silencing

Fatty acid elongase is another important gene in fatty acid metabolism regulated in different crops for the altered fatty acid composition. The down-regulation of FAE1 using antisense and hpRNA approach resulted in the reduction of erucic acid by 82 and 86% in Indian mustard respectively (Kanrar et al., 2006; Saheli et al., 2007). In the present study FAE1 silencing was measured by the change in OEP, or the amount of the substrate 18:1^Δ9^ that was elongated to 20:1^Δ11^. Levels of 20:1^Δ11^ in T_2_ seed transformed with the pJP1109 construct (FAE1 target) ranged from 2.2 to 15.1% in 12 transgenic events compared to an average in wild type level of 16.5% ± 0.3. A highly silenced line which has a single transgene insertion, determined by segregation ratios (3:1) of PPT forwarded to next generation (T_3_). Similarly, very low levels of 20:1^Δ11^ was observed in T_3_ seed ranged from 1.1 to 4.1%. The average 20:1^Δ11^ in T_3_ seed is 1.9 ± 1.0%, in contrast to 15.0% in control Columbia seed (Table 2). Thus, an 87% reduction was observed in the FAE1 activity with this miRNA approach. The fatty acid (18:1^Δ9^ and 20:1^Δ11^) averages of control and transformed lines were significantly different (*P *< 0.01). *Arabidopsis* ecotype Columbia has OEP value ranging 0.20–0.22, indicating that around 20–22% of 18:1^Δ9^ formed during fatty acid synthesis is subsequently elongated via the action of FAE1. In FAE1 silenced lines the OEP values ranged from 0.10 to 0.20 in T_2_ seed and 0.01–0.04 in T_3_ seed in contrast to 0.20 in control *Arabidopsis* ecotype Columbia (Figure 4B). Thus the activity of the FAE1 is reduced to 1% from 20% using the miRNA approach.

### FATB gene silencing

The AtFATB1 thioesterase is involved in the release of fatty acids from ACP, which are subsequently exported from the plastid and incorporated into eukaryotic lipids. In this experiment, FATB silencing was measured simply by comparing the levels of 16:0 in T_2_ and T_3_ seeds. Levels of 16:0 in T_2_ seed transformed with the pJP1110 construct (FATB target) ranged from 4.9 to 6.2% in 12 transgenic events compared to an average in wild type level of 8.0 ± 0.3%. A highly silenced line which has a single transgene insertion, determined by segregation ratios (3:1) of PPT was forwarded to next generation (T_3_). Similarly, low levels of 16:0 were observed in T_3_ seed ranged from 3.4 to 5.0%. The average 16:0 in T_3_ seed was 4.4 ± 0.5%, in contrast to 8.0 ± 0.3% in control Columbia seed (Table 2). Thus, an overall 55% reduction in 16:0 amount was observed in this study. In the FATB knock-out mutant (Gustavo et al., 2003) similar level of reduction (56%) in 16:0 was reported in *Arabidopsis*.

In summary, using the amiRNA approach, three important genes, i.e., FAD2, FAE1, FATB involved in seed oil metabolism were efficiently modulated. The efficiency of FAD2 and FATB silencing is matching with the well characterized mutant alleles. The amiRNA approach uses 21-bp single stranded RNA that target specific sequence offers distinct advantage with reduced risk of cross-silencing over intron-spliced hairpin or other approaches which use longer sequences (∼200). This precise silencing may be particularly useful for manipulating the seed oil composition of economically important oilseed crops.

## Conflict of Interest Statement

The authors declare that the research was conducted in the absence of any commercial or financial relationships that could be construed as a potential conflict of interest.

## Abbreviations

FAD2, fatty acid desaturase 2; FAE1, fatty acid elongase; FATB, fatty acyl-ACP thioesterase B, ODP, oleic desaturation proportion; OEP, oleic elongation proportion.
